# Continual Deep Learning for Time Series Modeling

**DOI:** 10.3390/s23167167

**Published:** 2023-08-14

**Authors:** Sio-Iong Ao, Haytham Fayek

**Affiliations:** 1 International Association of Engineers, Unit 1, 1/F, Hung To Road, Hong Kong; 2 School of Computing Technologies, RMIT University, Building 14, Melbourne, VIC 3000, Australia; haytham.fayek@rmit.edu.au

**Keywords:** deep learning, continual learning, sensor, time series, preprocessing, non-stationary, catastrophic forgetting

## Abstract

The multi-layer structures of Deep Learning facilitate the processing of higher-level abstractions from data, thus leading to improved generalization and widespread applications in diverse domains with various types of data. Each domain and data type presents its own set of challenges. Real-world time series data may have a non-stationary data distribution that may lead to Deep Learning models facing the problem of catastrophic forgetting, with the abrupt loss of previously learned knowledge. Continual learning is a paradigm of machine learning to handle situations when the stationarity of the datasets may no longer be true or required. This paper presents a systematic review of the recent Deep Learning applications of sensor time series, the need for advanced preprocessing techniques for some sensor environments, as well as the summaries of how to deploy Deep Learning in time series modeling while alleviating catastrophic forgetting with continual learning methods. The selected case studies cover a wide collection of various sensor time series applications and can illustrate how to deploy tailor-made Deep Learning, advanced preprocessing techniques, and continual learning algorithms from practical, real-world application aspects.

## 1. Introduction

Time series modeling is a challenging task in data mining and machine learning. Popular time series modeling tasks include classification, anomaly detection, regression, forecasting, and clustering. A time series is a sequence of measurements taken at various times. Spatial time series data refers to multiple time series data corresponding to different spatial locations. The spatial–temporal models face difficulties in addressing not only the short-term and long-term patterns but also the spatial patterns [1]. Time series datasets have the property of temporal ordering by nature [2]. Generally speaking, time series models have the capability to utilize the fact that observations closer together in time relate more closely. Time series modeling has many real-world applications like environment and traffic tasks, and successful modeling for the time series has become increasingly important. For example, wind time series forecasting is essential for the decision-making of electric system operators.

Time series analysis models can be divided broadly into time domain models and frequency domain models. The time domain model investigates the data with respect to time, while the frequency domain models focus the analysis on frequency instead of time [2]. Statistical models like autoregressive models and the moving average models are popular time domain models. Preprocessing operations like data cleaning, normalization, differencing, feature selection, etc., are also popular time domain models. For the frequency domain methods, mathematical models are employed to convert the time series data between the time and frequency domains. The Fourier transform is a popular, simple, and basic transforming tool for computing the frequency domain representation of a time series. Other popular preprocessing of frequency domain are wavelet transformation (WT), empirical mode decomposition (EMD), etc. 

Over the last decade, smart sensors have been deployed on a very large scale, and huge amounts of continuous data have been generated [3]. Machine learning (ML) methods can extract valuable information from datasets and have been widely employed for sensory data in the industry, such as in the sensing and condition monitoring fields [4]. In the knowledge discovery process, feedback is generated at each iteration with the goal that further improvement can be achieved [5]. Deep Learning (DL), a particular type of machine learning algorithm with multi-layer structures for processing higher-level abstractions from the input dataset [6], is very well suited for very large datasets, as most of its layer computations can be implemented in parallel and distributed computing techniques can be applied easily. Deep Learning models have been shown to perform satisfactorily for many time series analysis tasks like forecasting. 

Deep Learning (DL) has been developed from traditional neural networks with large-sized deep structures since 2006. Compared with traditional machine learning, DL learns through a general-purpose learning procedure of multiple levels of representation and identifies features automatically [7]. In each layer, the representation is obtained from the representation of the previous layer, involving updating process with a back-propagation algorithm. Different from the more traditional ML methods, the addition of more layers in the Deep Learning model can further enable the deep network to cope with scenarios of increasing complexity, thus leading to improved generalization. A multi-layer learning structure enables very high performance in complex situations like video, speech, classification problems, and multi-sensor aggregation. 

Deep Learning models have been shown to perform satisfactorily for many time series analysis tasks. For example, Deep Learning has proven excellent in human activity recognition (HAR) tasks, where wearable sensors can connect people with the cyber–physical system through HAR [8]. Deep Learning has also been employed for tipping-point prediction, with performance better than traditional early warning systems [9]. Nevertheless, the reliability of these forecasting methods is not guaranteed [10]. Deep Learning methods may also face the overfitting problem. Common preprocessing methods like smoothing, transformation, and estimation can remove the noise in time series signals in advance and improve the overall performance of the time series models. The performance of Deep Learning models may improve with preprocessing of time series inputs, usually on conditions that the data distribution at test time is similar to that at training time. 

When building ML models, it is usually assumed that the distribution of the data is stationary. When the statistical properties of a time series do not depend on time, the time series is called stationary [2]. It is possible that a time series is stationary in terms of one characteristic while non-stationary for another characteristic. Mathematically, a time series TS(*y_t_*) is defined to be (weakly) stationary if all time *t*:

*E*(*y_t_*) = *E*[(*y_t−1_*)] = *μ*,

*Var*(*y_t_*) = σ^2^ < *∞*,

*Cov*(*y_t_*, *y_t−k_*) = γ(*k*),

where the expected value *μ* is represented by *E*(.), the variance σ^2^ by *Var*(.), and the covariance γ by *Cov*(.), respectively [11]. If the stationary conditions are no longer true, the non-stationary behaviors may pose significant difficulties for time series applications like remote sensing [12].

In many real-world applications, the stationarity of the datasets may no longer be true. There are four basic components that can cause the non-stationarity in some time series. These four components are trend, seasonal, cyclical, and irregular components [13]. The trend component refers to long-term increases or decreases over time, with examples like long-term population growth. The seasonal component refers to the existence of seasonality patterns, like, for example, time series with yearly, quarterly, or monthly patterns. The cyclical component refers to the patterns of ups and downs of a time series over the duration of a cycle. The economic time series data of economic growth and then economic recession may have a cyclical component. The irregular component is due to unpredictable factors and does not repeat with particular patterns. For some time series data that exhibit a simple trend, seasonal, and cyclical components, DL methods may be deployed directly with satisfactory results, as seen in the first part of Section 2. For some more complicated sensor time series datasets, advanced preprocessing tools may be needed, as seen in the second part of Section 2. Among the time series datasets that can not be handled well with both advanced preprocessing and DL methods, some may fit the scenarios for the deployment of CL methods, which are described in detail in Section 3.

Continual learning is a paradigm of machine learning that may handle some of the non-stationary situations while imitating the capability of human intelligence to handle new situations from the old experiences learned. CL algorithms are developed to mimic human intelligence that will rarely forget all of the learned information, as the natural system will only gradually lose the learned information [14]. On the other hand, traditional neural networks (NNs), while mimicking human cognition, lack this ability and face catastrophic forgetting (CF). If Deep Learning methods are naively employed in CL tasks, learning on shifted distribution may lead to CF problems. The capability of how to learn continually is one of the biggest unsolved issues in ML [15], while forgetting learned knowledge is the key obstacle to continual learning [16]. 

This survey focuses solely on real-world practical time series applications with DL and CL. Lange et al. [17] presented a survey on CL about the stability–plasticity trade-off and focused on the problems of classification only. The motivation for them to limit the discussion to classification alone is that NN has been very well-established for classification tasks. In the survey [18], it was highlighted that the most recent surveys usually covered continual learning partially, like biological underpinnings [19,20,21,22], visual classification tasks [23,24,25,26], NLP tasks [27,28], and RL [29]. Wang et al. [18] talked about only five tasks of practical CL applications, i.e., object detection, semantic segmentation, conditional generation, reinforcement learning, and NLP. None of the above-mentioned recent CL surveys focused on time series analysis tasks like time series forecasting. The focus of this survey is solely on the current application case studies of DL for sensor time series datasets. It provides a summary of the difficulties that the DL faces in deployment for real-world sensor applications and the advanced preprocessing techniques for how to address some of these practical difficulties. There are some cases that typical DL approaches, together with advanced preprocessing techniques, can handle well. Nevertheless, in some other time series application cases, continual learning is found to be suitable for deployment. The topic of deploying CL for sensor time series modeling has not been systemically covered in these previous surveys.

This paper is organized with the following sections, covering the recent time series applications of Deep Learning and continual learning. In Section 2, firstly, the focus is on the current advances in deploying Deep Learning methods for sensor time series modeling. Then, the recent sensor case studies that still require data preprocessing techniques for DL modules are highlighted. In Section 3, a summary of the sensor time series datasets and environments that suit the deployment of continual learning methods is undertaken.

## 2. Advances in Deep Learning Methods for Time Series Modeling

Deep Learning is capable of modeling the complex non-linear relationships among the variables, while traditional neural network needs to assume that all input vectors are independent, which may lead to its ineffectiveness for sequential data prediction [30]. Comparing DL with the conventional time series methods [13,31], it is found that Deep Learning models can give better representation and classification. Cai et al. [31](2019) investigated the day-ahead multi-step load prediction of commercial buildings with RNN and CNN against autoregressive integrated moving average with exogenous inputs (ARIMAX), a popular traditional time series method for the time series modeling of load forecasting. The results show that the CNN approach with a direct multi-step procedure can perform better than the seasonal ARIMAX by a 22.6% improvement in prediction accuracy. This illustrated that the Deep Learning hierarchical structure may have the capability to handle data-dependent uncertainty better. It is also shown that the long-term trends can be explored better when the preprocessing tool of the moving averages method is deployed for smoothing the short-term fluctuations. Mahmoud and Mohammed [13] presented a survey of Deep Learning models, such as CNN, RNN, LSTM, GRU, deep autoencoders (AEs), restricted Boltzmann machines (RBM), deep belief networks (DBNs), in the time series forecasting of electricity load and price, solar power, and finance, with comparison results showing that DL performs better than classical methods.

**Figure 1:**
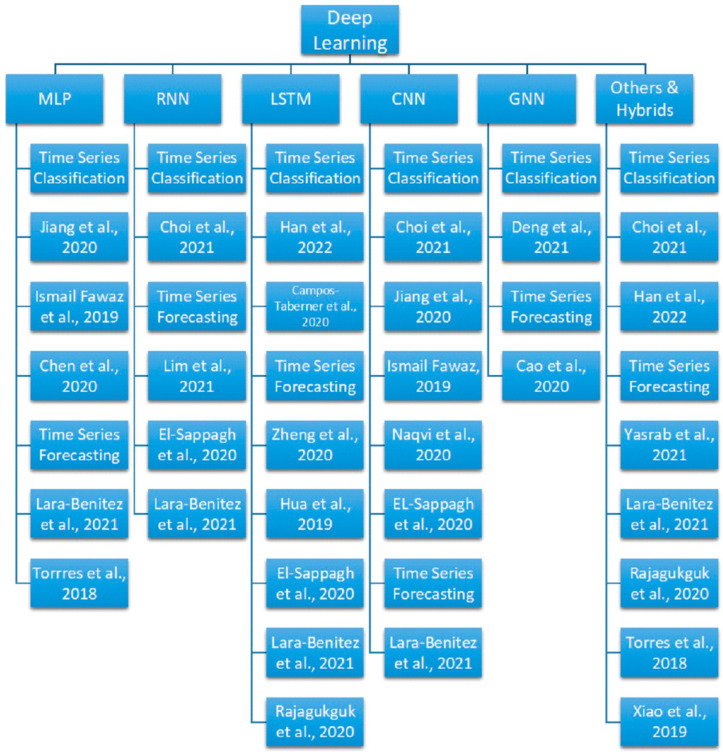
Tree diagram for grouping the popular Deep Learning methods for sensor time series classification and forecasting tasks covered in this survey [30,32,33,34,35,36,37,38,39,40,41,42,43,44,45,46,47,48] (note: if a paper uses two methods separately with similar satisfactory results, the paper will be listed under both groups).

After discussing the advantages of deploying DL methods with time series data, the following Table 1 shows a synthetic summary of the advances in Deep Learning techniques for real-world sensor time series applications, followed by a detailed description of the corresponding methods, motivations, and advantages. The following Figure 1 shows the tree diagram grouping the popular Deep Learning methods for sensor time series classification and forecasting tasks covered in this survey.

Multi-layer perceptron (MLP), recurrent neural network (RNN), Long Short-Term Memory network (LSTM), convolutional neural network (CNN), graph neural networks (GNN), deep belief network (DBN), autoencoder (AE), and neural networks with an attention mechanism are popular DL methods that have been used by the authors in this survey.

### 2.1. Multi-Layer Perceptron

MLP is the most conventional and simplest Deep Learning structure and is fully connected (FC) between layers. All the neurons in one layer are connected to every neuron in the nearby next layer. This means that each time series record is assigned its own weight, and the temporal information is not utilized [35]. During the training phase of the MLP, the weight between the neurons of two nearby layers can be estimated by the minimization of cost function through gradient descent optimization. The computing of the gradient of the cost function is used in the back-propagation algorithm. 

Jiang [34] conducted a comprehensive evaluation of the Deep Learning methods (MLP, CNN, and ResNet) against a conventional machine learning method—the nearest neighbor. The 1-NN classifiers are deployed with eight different distance measures. A total of 128 univariate time series datasets about image, motion, and sensor from the UCR Time Series Classification Archive [49] were tested for the classification performance of the models. The experimental results showed that the Deep Learning methods could have better performance than the nearest neighbor, but their difference is not significant when appropriate distance measures are deployed for the nearest neighbor based on the types of the time series datasets.

Ismail Fawaz et al. [35] comprehensively evaluated the performance of three popular structures of Deep Learning for time series classification (TSC), i.e., MLP, CNN, and Echo State Network (ESN). The Deep Learning models for TSC tasks can be classified as generative and discriminative approaches. The essential structure of ESN is the reservoir, a sparsely connected random RNN with random initialization in the hidden layers. Jaeger and Haas [50] invented the ESN in 2004 for time series forecasting for wireless communication tasks. The evaluation was conducted with the 85 univariate time series datasets of the UCR/UEA archive [51] and 13 MTS datasets from Baydogan’s archive [52]. For univariate time series applications, a one-dimensional CNN model is used, and the filter can be viewed as performing a non-linear transformation for the time series. For multivariate time series, the dimensions of the filter are set to be the number of dimensions of the time series. For time series classification, the final layer is the discriminative layer which can assign probability distribution for the class variables of the time series concerned. The results supported that end-to-end Deep Learning is able to reach state-of-the-art performance for TSC tasks.

Chen et al. [41] presented a Deep Learning method based on MLP for equipment reliability prediction with sensor time series data. Condition monitoring for equipment and its maintenance prediction is important for smart manufacturing. It is desirable to conduct maintenance before the machine failure happens. The experiment was conducted with a reliability test of a cylinder in the small trolley of vehicle assembly plant. As it takes longer to complete each operation for an aging cylinder, the cylinder operation duration can serve as the time series inputs for the Deep Learning method. The results show that the Deep Learning method can perform much better than conventional machine learning methods.

Lara-Benitez et al. [45] compared the performance of seven popular Deep Learning algorithms against twelve time series forecasting tasks. Time series forecasting is the process of utilizing relevant historical time records to determine future values. When deploying CNN for time series modeling, causal convolutional filters are used to ensure that forecasting is made only based on past information in the time series [53]. The same as standard CNN, temporal CNN also shares the same assumption that the relationships among the time series variables are time-invariant. In attention mechanisms, the temporal features of the time series signals can be aggregated with the dynamical generation of weights by the attention layers. The attention network can pay more attention to the significant historical event, no matter how far back it is in the key-value lookup window. The Deep Learning models are MLP, four models of recurrent networks (Elman RNN, LSTM, Echo State Network, GRU), and two convolutional networks (CNN and Temporal Convolutional Network). The twelve public datasets cover time series applications like finance, industry, solar energy, tourism, traffic, and internet traffic. The evaluation results showed that LSTM performs the best, while CNN can also make accurate and stable forecasting with less computational requirements. Another performance comparison of Deep Learning methods can be found in Torres et al. [54], making a summary of the satisfactory time series forecasting applications of the popular Deep Learning models (ENN, LSTM, GRU, BRNN, MLP, CNN, and TCN) in the sectors like energy, environment, finance, health, industry, and image. The practical tip on how to set hyper-parameters has also been covered. The four main types of hyper-parameter optimization methods are trial-error, grid, random and probabilistic.

Torres et al. [47] proposed a Deep Learning approach that was based on the H20 package written in R with the grid search method for hyper-parameter optimization and implemented in the Apache Spark cluster environment. As the manufacturing cost for solar panels decreases, solar energy is becoming much more popular. It is predicted that 30% of the total electricity sources will be from solar energy in Australia by the year 2050 [55]. Factors like cloud cover, rainfall, solar radiation, and temperature can affect the generation of solar energy significantly and make the forecasting of solar energy generation difficult. The dataset is the two-year time series of PV power with 30 min observation interval. Its performance was compared with pattern-sequence-based forecasting (PSF) and conventional neural networks. The experimental results supported that the Deep Learning approach is very suitable for the solar energy forecasting task.

### 2.2. Recurrent Neural Network

RNN is designed with a state for the information at time steps such that it can handle sequence data element-wise [56]. As it can maintain the dependencies among the time series elements, RNN has gained success in different fields such as image captioning, machine learning translation, speech recognition [57], and weather forecasting [58]. Elman Neural Network (ENN) is an RNN model with a new hidden layer called the context layer, which makes it capable of data sequence prediction. Nevertheless, RNN faces difficulties due to the vanishing and exploding gradient problems during its training process. This disadvantage of the vanishing gradient problem can be amplified when considering long-range dependent relationships. 

Choi et al. [32] focused on the frontier time series anomaly detection methods and described how the methods model the interrelationship among variables and how the temporal context was learned. Time series anomaly detection is about the identification of unexpected events from the time series data. Because of their learning capability for large-scale sequences, Deep Learning methods contribute significantly to multivariate time series anomaly detection. Nevertheless, most of these methods have been developed for very specific applications, and sufficient domain knowledge is necessary [32]. Benchmark datasets of the water treatment test-bed, the water distribution pipelines, and the Mars Science Laboratory rover were used to compare the performance of the methods (RNN, CNN, hybrid, and attention). The result highlights that there is no single Deep Learning method that can fit universally with all the time series tasks. Even though emerging Deep Learning methods show encouraging results for multivariate time series anomaly detection, most of these methods cannot explain the relationships among the sensors and thus have limited ability to explain the deviations of the anomalous events.

Lim and Zohren [42] summarized the recent trends of Deep Learning models and hybrid models. Hybrid methods combining quantitative time series models with Deep Learning may address these limitations and improve the overall forecasting performance [42]. A hybrid model may be able to incorporate domain experts to feed the Deep Learning model with prior information while addressing the issues associated with small datasets and overfitting. Exponential smoothing RNN (ES-RNN) serves as a good example of how the exponential smoothing module can help the RNN by addressing the non-stationary trends of the time series by winning the M4 competition [59].

El-Sappagh et al. [44] presented an ensemble Deep Learning approach of stacked CNN and Bidirectional LSTM (Bi-LSTM) models for AD progression detection. The temporal information from sensor monitoring of chronic disease can be helpful for progression detection, while the progression of chronic Alzheimer’s disease (AD) may be delayed if it can be predicted at an early stage. The data of AD patients are heterogeneous multimodalities. Bi-LSTM is a special type of LSTM that can explore the dependencies of both previous and future states [6]. For time series prediction with Bidirectional LSTM, once a forecast is available in training, this value can be utilized for the subsequent readjustment process. This may result in a deeper understanding of the context concerned. The model can handle the fusion of the five different types of time series data along with background knowledge. The stacked CNN and Bi-LSTM models are capable of extracting both local and longitudinal features of the modality, and the system can make predictions based on these resultant features. The time series data from the Alzheimer’s Disease Neuroimaging Initiative were used, and the results support the superior performance of the proposed ensemble Deep Learning approach. For traditional methods to succeed in structural health monitoring, suitable preprocessing of the raw sensor time series data is nearly always needed. Dang et al. [60] applied four popular Deep Learning models, i.e., MLP, LSTM, 1D CNN, and CNN, for structural damage detection with raw time series data. No feature engineering procedure like extracting structural characteristics was performed. Three different structures covering small to large structures were tested. The experimental results support that 2D CNN is the most reliable, even though the computational time of 2D CNN is also the highest among the models.

### 2.3. Long Short-Term Memory

Hochreiter and Schmidhuber [61] designed a new RNN model, called Long Short-Term Memory (LSTM), with an internal memory unit and gate mechanism, for addressing the issues of vanishing and exploding gradient during RNN training. A self-feeding loop is used in the inner layers of LSTM that can learn time-based correlations such that knowledge from previous inputs can be used in the analysis of the present inputs. The three gates implementation [6] of LSTM can reduce the effects of the vanishing gradient problem faced by RNN. The three gates are the forget gate, update gate, and output gate. The forget gate is to determine what information should be kept or not. The update gate is to determine what new information should be utilized for updating the memory state. The output gate is to determine the output value that will serve as the input in the next hidden unit. This design can enable the keeping of longstanding related information while forgetting unrelated information. Thus, LSTM has the capability to process the long-term dependencies of information in temporal datasets and is deployed for applications like speech recognition, traffic prediction, and weather forecasting. The Gated Recurrent Unit (GRU) is designed with a less complex RNN structure than LSTM [62] but can also remember useful information and explore the long-term dependencies of the variables. A GRU has only two gates, i.e., the update gate and the relevance gate. The update gate is to determine if the memory state is to update or not. The relevance gate is to determine how related the previous memory state is for computing the next candidate. As GRU has only two gates, it requires fewer parameters and computational time than LSTM [63] while still keeping the capability for handling very long-range relationships among the time series.

Han and Sanchez-Azofeifa [36] investigated the leaf and wood terrestrial laser scanning (TLS) time series classification with Fully Convolutional Neural Network (FCN), LSTM-FCN, and Residual Network (ResNet). CNN has been found to be capable of performing time series classification satisfactorily, as its multiple filters can produce multiple discrimination features for classification from the temporal inputs [35]. It is also found that the combined LSTM-FCN method can further improve the time series classification results by FCN [64]. TLS point cloud data are useful for classifying the leaf and woody components, and thus the leaf area index and wood area index. The TLS time series point clouds were observed from seven broad-leaved trees (Ulmus americana). The experimental results showed that all three models work better with multivariable time series than with univariable time series. The three models can also work better than previous methods, and all these three models produce similar performances on the testing time series.

Campos-Taberner et al. [37] investigated the interpretability of a 2-layer Bi-LSTM network for the classification of land use in Valencia, Spain, with Sentinel-2 time series data. Added-noise permutation procedure was employed in both temporal and spectral domains for evaluating the impact of different spectral and temporal features on the accuracy rate. The experimental results showed that the overall accuracy of 98.7% achieved by the proposed method is better than other classification approaches. The proposed system can also show the relevance of predictors and highlight that the red and near-infrared Sentinel-2 bands contain the most helpful information, while the summer time series data is most useful among the temporal information.

Zheng and Huang [39] deployed the Deep Learning LSTM network for the forecasting of traffic flow time series. In an intelligent traffic system, the accurate forecasting of traffic flow may help to reduce urban congestion. The traffic flow time series from OpenITS with 5 min intervals in ten days was used, and its performance was evaluated with the traditional statistical method (ARIMA) and the conventional machine learning method (BPNN). The results support the superior prediction accuracy of the LSTM model, with the mean absolute percentage errors of ARIMA, BPNN, and LSTM at 20.97%, 9.06%, and 4.82%, respectively.

Hua et al. [40] deployed the Random Connectivity LSTM (RCLSTM) for traffic prediction and user mobility of telecommunication problems in order to reduce the computational requirement of LSTM. In RCLSTM, the neurons are connected stochastically, and some sparsity can be achieved in this random sparse graph model to reduce computing time. RCLSTM is found to be suitable for latency-stringent tasks like traffic time series. Another similar task is the evaluation of the traffic time series data from the GEANT backbone network by Uhlig et al. [65]. The experimental results showed that the forecasting performance of RCLSTM is similar to the traditional LSTM while successfully reducing the computational time.

Rajagukguk et al. [30] investigated the prediction performance of solar irradiance and photovoltaic (PV) power with four Deep Learning models against conventional machine learning methods. As solar energy is renewable, solar photovoltaics has gained popularity in the generation of electricity. For the PV prediction, the popular input time series data are the temperature, humidity, and wind speed. The experimental results confirmed that all four Deep Learning models, i.e., RNN, LSTM, GRU, and CNN-LSTM, can perform the prediction better, with CNN-LSTM achieving the best performance but also needing the longest training time period. In the hybrid model CNN-LSTM, the CNN layers can perform the feature extraction of the input time series, while LSTM for sequence prediction. CNN-LSTM has been designed with the capability to handle temporal prediction tasks and applied in many real-world applications. Experimental results like [66] also supported the consistently better performance by CNN-LSTM over LSTM for precipitation prediction.

### 2.4. Convolutional Neural Network

CNN is another popular neural network for time series modeling. CNN is a DL method of interconnected feedforward network architectures consisting of a sequence of convolution, pooling, activation layers, and, finally, fully connected layers [67]. Filtering operation is performed in the convolutional layers through a feature map. Local confluences of features are identified from the preceding layer through discrete convolution. Thus, this type of model becomes known as a convolutional neural network. The pooling layer is for reducing the input size and avoiding overfitting. The input for the last dense layer is the flattened features from the convolutional and pooling layers, and the forecasting is made in this dense layer. 1D CNN can be deployed for simple applications, while more sophisticated models, like CNN-Net, Enoded-Net, and CNN-LSTM, have more advanced structures like larger kernel size and denser layers.

Naqvi et al. [38] deployed a CNN model for detecting changes in gaze from the face and left- and right-eye images. Driver time series data is helpful for the real-time classification of normal and abnormal driving and, thus, for reducing aggressive driving. The near-infrared (NIR) camera sensor is employed here to construct a large database of driver facial emotion and gaze. The Dlib facial feature trackers were employed to identify the region of interest (ROI) before feeding to the CNN model. The experimental results supported the outstanding performance of the CNN model.

### 2.5. Graph Neural Network

GNN is a neural network that processes the data with representation in graph domains, like in chemical compounds, images, and web [68]. Graphs can be classified as cyclic, directed, undirected, or a combination of these three. GNNs have been found useful for applications including chemistry, citation networks, environmental condition forecasting, molecular biology, physics, and social networks. A few DNN topologies may indeed be viewed as GNN, for example, with CNN considered as a GNN with graphs of pixel-per-grid grids.

Deng and Hooi [33] developed a Graph Deviation Network (GDN) model for discovering the relationship graph among the sensors and for detecting deviation from normal patterns. Graph neural networks can model the complex patterns in data of graph structure, with the node state influenced by its neighbor note states. Velickovic et al. [69] developed the graph attention network (GAT), which employed the attention function for evaluating the various weights for various neighbors during aggregation. A difficulty for a typical GNN model is that the graph structure is needed as an input, while this structure may not be known in advance. The proposed system consists of sensor embedding, graph-structured learning, graph-attention-based forecasting, and graph deviation scoring. The system is tested with two large-scale sensor time series datasets of water treatment systems (SWaT and WADI). The experimental results supported the performance of the proposed system, while the interpretable output can assist users in better understanding and localizing anomalies.

Cao et al. [46] presented Spectral Temporal Graph Neural Network (StemGNN) with Graph Fourier Transform (GFT) for capturing inter-series correlations and Discrete Fourier Transform (DFT) for temporal correlations. A difficulty for multivariate time series is the complexity involving both the temporal correlations of intra-series and the correlations of inter-series simultaneously. The spectral representations obtained with GFT and DFT enable the Deep Learning modules to have clear enough patterns for the analysis. The experimental results from ten real-world time series public datasets (from energy, electrocardiogram, and traffic sectors) confirm the performance of the proposed StemGNN.

### 2.6. Others and Hybrids

Hinton and Osindero et al. [70] developed the first model and training algorithm for a deep belief network (DBN) in 2006. DBN has a hierarchical structure with a large number of stacked restricted Boltzmann machines (RBMs) and utilizes a greedy layer-by-layer learning approach with fine-tuning. The RBMs can efficiently extract the features for the initialization of feedforward neural networks, hence improving the network generalization capability. In each RBM, there is both a visible layer and a concealed layer consisting of neurons. While the RBM’s layers are interconnected with each other, the units among each layer are not. The updating of the network parameters can be done with a SoftMax classifier.

Autoencoder utilizes a feature learning paradigm that can learn para-metric maps directly from inputs to corresponding representation [71,72]. An AE consists of an encoder and a decoder. The encoder is for feature extraction, while the decoder is for mapping the feature space back to the input space. The structure of the encoder and decoder is an input layer, hidden layers, and an output layer. Back-propagation algorithm is deployed for updating the weights of the hidden layers. Deep AE can serve as a data-driven approach for learning feature extraction automatically.

Neural networks with an attention mechanism have succeeded in time series applications [73]. These networks can dynamically control the mapping from the inputs to the outputs intelligently with other knowledge of the task. The transformer model is based on an encoder–decoder architecture of these networks. The input is fed to the encoder, which consists of a stack of encoders. Then, the transformer can generate the output based on the encoded input and previous outputs in the decoder as well. A clear advantage of transformers is the access to any points in the past, no matter how far their distances are, leading to the capability to discover the long-term dependencies among the time series data.

Yasrab et al. [43] designed a Generative Adversarial Network (GAN) for forecasting the expected growth of the plant. Plant phenotyping is the investigation of plant trait growth and other quantitative parameters and may be automated with the help of Deep Learning. GAN is usually developed from CNN and is formed with two networks, i.e., the generator and the discriminator. Both networks can learn together by competing with each other to generate new examples of synthetic data. Segmentation masks of shoot and root were generated to predict the plant system in the future. Two public datasets (*Arabidopsis* and *Brassica rapa* plants) were used. The evaluation results illustrated that the proposed system can reach the level with annotation by an expert.

Xiao et al. [48] developed a Convolutional Long Short-Term Memory (ConvLSTM) model to utilize the spatiotemporal correlations of sea surface temperature (SST) for its short and mid-term prediction. In the global ocean, SST has a significant influence on the marine ecosystem. SST time series data from 36-year observations by satellite was used to evaluate the ConvLSTM against the persistence model, support vector regression model, and LSTM models. When handling spatiotemporal data by fully connected LSTM (FC-LSTM), it is noted that the spatial correlations can be lost [66]. To address this issue, the ConvLSTM replaces the FC-LSTM matric multiplication with convolution operation in the transitions. This is to ensure that the model can explore both spatial and temporal correlations. The experimental results confirmed that ConvLSTM performs better than other methods for the one-to-ten-day-ahead prediction of SST time series.

### 2.7. Advanced Preprocessing and Deep Learning Applications

Feature engineering can be very important in the data preprocessing stage before feeding data to the Deep Learning models by significantly reducing the computational requirements for unnecessary features. Its importance is highlighted in the work of Elsayed and Thyssens et al. [74], which evaluated the time series forecasting performance of eight Deep Learning methods against the traditional machine learning method—the Gradient Boosting Regression Tree (GBRT). External features were utilized for the target values, and data flattening can obtain the one input instance for GBRT. The evaluation was conducted with nine datasets covering air quality, electricity, finance, solar energy, and traffic. The results showed that the window-based transformation could enable GBRT to achieve the best forecasting performance over the Deep Learning models, covering various types like matrix factorization, RNNs, LSTMs, and bi-directional LSTM models. It is shown that simpler machine learning models with efficient feature engineering can outperform the frontier Deep Learning methods without feature engineering. Dablander and Bury [9] highlighted the importance of preprocessing by showing that the DL model cannot extract enough relevant features for classifying the stationary AR(1) processes without detrending or with a Gaussian filter. The results showed that the method might learn unique features related to a Lowess filter instead of the relevant features of the system near the bifurcation. Thus, careful planning is needed when deploying the preprocessing models. 

**Figure 2:**
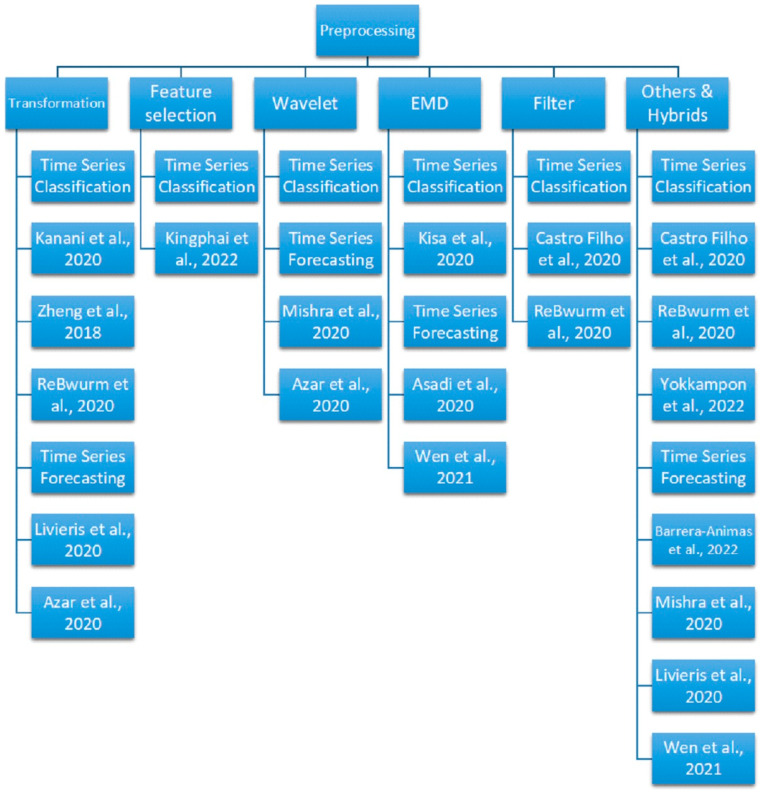
Tree diagram for grouping the popular preprocessing methods for sensor time series classification and forecasting tasks covered in this survey [1,8,10,58,75,76,77,78,79,80,81,82,83] (note: if a paper uses two methods separately with similar satisfactory results, the paper will be listed under both groups).

The following Table 2 highlights the most recent case studies that advanced preprocessing is still needed, along with the Deep Learning methods, for modeling different popular types of sensor time series. In Table 2, a synthetic summary of the application fields, the advanced preprocessing techniques, the DL methods, accuracy, and brief application details is presented, followed by a more detailed description of the corresponding methods, motivations, and advantages. The following Figure 2 shows the tree diagram for grouping the popular preprocessing methods for sensor time series classification and forecasting tasks covered in this survey.

Kanani and Padole [75] presented a preprocessing framework that can increase the electrocardiogram (ECG) classification accuracy of the Deep Learning methods significantly, with a higher than 99% accuracy rate. ECG time series signals are very efficient for the monitoring of cardiovascular health, with abnormal heartbeats detected from the ECG patterns. The labeled MIT-BIH Arrhythmia dataset, which has ECG time series signals of five different classes, was tested. The proposed preprocessing steps include the squeezing and stretching of the signal along the time axis and the amplifying and shrinking of the amplitude of the signal. It is shown that these transformations do not change the characteristics of the signals and can be regarded as completely lossless transformations here.

Kisa et al. [76] deployed an adaptive preprocessing method with empirical mode decomposition (EMD) was proposed to handle this nonstationary time series, as the surface electromyography (sEMG) time series is nonstationary and nonlinear. sEMG can be employed to measure the electrical activity of human muscles. The recognition of human movements is useful for applications like human–computer interaction (HCI). The sEMG hand gesture time series data was obtained with a sensor device of surface bipolar electrodes with 30 healthy volunteers. EMD can serve as a non-linear filter to decompose the input time series into several intrinsic mode functions (IMFs). The outputs of the IMFs were then fed into the Deep Learning model CNN based on Residual Networks (ResNet) for gesture classification. It is found that the IMFs can improve the validation accuracy of the original time series from 94.22% to a maximum of 99.73%.

Zheng et al. [8] investigated the segmentation and transformation methods for their effectiveness in data preprocessing for Deep Learning algorithms. In HAR tasks, segmentation methods are essential as raw inertial time series can have very large fluctuations. Five segmentation options of five different segment lengths were evaluated with four transformation methods, i.e., raw plot, multichannel, spectrogram, and spectrogram with shallow features. The experiment was conducted with datasets of eight daily activities from wearable sensors, car workshop maintenance activity data, etc. The results showed that the classification accuracy increases along with an increase in segment length, and the multichannel method can perform the best for the HAR tasks. The overall accuracy is 97.2%, which is better than many other machine learning models.

Castro Filho et al. [77] applied a two-stage noise scheme for preprocessing SAR time series, as there are inevitable noises like speckles for Synthetic Aperture Radar (SAR) images. SAR sensors can be employed for mapping the rice-growing regions and constructing continuous time series data. The SAR data is very useful for monitoring short phenological stages and raising the classification capacity. 3D-Gamma filter was used to eliminate the speckle, and the method of Savitzky and Golay [84] was employed to smooth the time series. The processed time series was fed to two Deep Learning methods, the Long Short-Term Memory model and the Bidirectional LSTM model, for mapping rice crops with SAR sensor time series from West Rio Grande do Sul (Brazil). The results were compared with conventional machine learning models, with BiLSTM showing the best performance in the McNemar test. 

ReBwurm and Korner [78] investigated the effectiveness of Deep Learning models for the classification of crop type based on raw and preprocessed Sentinel 2 satellite time series data. With the advance of remote sensing technologies, the amount of the Earth observation time series data has been greatly increasing over recent years. While many traditional models for remote sensing applications require preprocessing and feature extraction, ReBwurm and Korner [78] checked if the current Deep Learning methods are able to utilize the raw time series data directly without data preprocessing. Atmospheric correction, filtering of cloud temporal observations, focusing on vegetative periods, and masking of clouds, which are typical preprocessing methods for satellite time series, have been applied. Additionally, 1D-convolutions, recurrent neural networks (RNN), and the self-attention model of the encoder architecture of the Transformer network [85] are deployed and evaluated. The results show that self-attention and RNN can perform better for raw data, while the preprocessing process can improve the results for all three methods satisfactorily.

Kingphai and Moshfeghi [79] employed seven Deep Learning models, i.e., convolutional neural network (CNN), Stacked Gated Recurrent Unit (SGRU), Bidirectional GRU (BGRU), BGRU-GRU, LSTM), Bidirectional LSTM (BiLSTM), and BiLSTM-LSTM, for classifying mental workload (MWL) levels from electroencephalography (EEG) time series signals. MWL can lead to a better understanding of human performance in complex environments. EEG time series can be utilized for classifying the mental workload level of a human subject. The dataset STEW contains signals from 48 subjects in the resting and working states. Preprocessing is needed because there is usually noise in the EEG signals. The independent component analysis based on ADJUST (ICA-ADJUST) by Mognon et al. [86] was found to be the most effective preprocessing tool for this dataset by Kingphai and Moshfeghi [87]. Kingphai and Moshfeghi [79] extracted four groups of features (frequency domain, time domain, linear domain, and non-linear domain) for the Deep Learning models. The results showed that BiLSTM-LSTM performed best with 94.75% accuracy for classifying resting vs. working states, while BGRU-GRU was best for classifying low vs. moderate vs. high MWL levels with 83.03% accuracy.

Yokkampon et al. [80] deployed multi-scale attribute matrices as the preprocessing tool for transforming the multivariate time series to develop a multi-scale convolutional variational autoencoder for unsupervised anomaly detection of multivariate sensor time series datasets. The attribute matrices can utilize the pair-wise inner product of the time series among segments and effectively characterize system states of the time series. The identification of anomalies is about detecting data points that deviate significantly from their expected values. There are three types of time series anomalies: (1) point anomalies, which refer to outlier points; (2) contextual anomalies, which differ significantly from typical points of the same context; and (3) collective anomalies, which refer to the existence of a subset of time series data points with a significant difference from the other points in the whole dataset [88]. Anomaly detection can be employed in many real-world cases, like fraudulent transaction detection, sensor network fault analysis, and abnormal equipment monitoring. The proposed model was tested with four publicly available benchmark datasets: the time series data by the Australian Centre for Remote Sensing, the Wafer time series dataset of semiconductor microelectronics fabrication, the Emotiv EEG Neuroheadset time series dataset, and Opt handwriting dataset. The experimental results show that the model performed better than other baseline methods.

In the investigation by Barrera-Animas et al. [58], before feeding the time series data into the DL models, the feature selection process was achieved with the correlation matrix (CM), which is computed with the Pearson correlation coefficient for the 43-dimensional datasets. Rainfall prediction is complicated because of its nonlinear characters. Spatial information like latitude and longitude and atmospheric information like humidity, pressure, temperature, and wind speed may be utilized for the forecasting models. Barrera-Animas et al. investigated the effectiveness of Deep Learning models, including LSTM, Stacked-LSTM, and Bidirectional LSTM, with the conventional machine learning (ML) model XGBoost, and also automated machine learning (AutoML) with the TPOT tool [89], which can be regarded here as an ensemble of ML models. OpenWeather data of five UK cities from 2000 to 2020 were evaluated. Highly correlated features will be eliminated. The experimental results highlighted that the Stacked-LSTM with two hidden layers and the Bidirectional LSTM could obtain the best performance in rainfall forecasting. 

Mishra et al. [81](2020) evaluated the wind predictions with five Deep Learning methods against three data preprocessing tools. The five methods are the attention mechanism (Attention) of sequence-to-sequence encoder–decoder architecture [90], deep convolutional network (DCN), deep feed Forward (DFF), recurrent neural network (RNN), and LSTM. The dataset is the time series of the temperature and wind power variable. Discrete wavelet transformation and fast Fourier transformation (FFT) are employed to transform the time series dataset before feeding to the Deep Learning models, while inverse transformation was applied to the DL model outputs before making predictions. The experimental results showed that Attention and DCN work the best with wavelet and FFT, while some other models work better with no need for data preprocessing.

Livieris et al. [10] proposed a preprocessing framework to further improve the efficiency and reliability of the Deep Learning methods. Iterative transformations and augmented Dickey–Fuller test were applied to the time series data for obtaining stationary processed time series data before feeding to the Deep Learning model. The Ljung–Box Q test was employed to check the autocorrelation of the model’s errors. Time series data from energy section, stock market, and cryptocurrency were tested, and the experimental results showed that the proposed preprocessing framework could enhance the efficiency, accuracy, and reliability of the Deep Learning LSTM and CNN-LSTM models considerably. Livieris et al. [91] continued their work on the investigation of the preprocessing framework for Deep Learning models. On top of their previous focus on the transformation of non-stationary time series to stationary by differencing the time series, the raw time series is now subject firstly subject to the exponential smoothing method, which can transform the raw data to a de-noised version. This process is to increase the quality of the time series data and thus improve the prediction capability of the Deep Learning model CNN-LSTM. The experimental results with cryptocurrency, energy, and stock markets confirmed that the preprocessing framework could significantly achieve its objective of further improvement for the Deep Learning models.

Asadi and Regan [1] employed the time series decomposition method to obtain the short-term, long-term, and spatial patterns in the proposed preprocessing framework. The short-term patterns of the spatial time series were explored with the fuzzy clustering method, which can group neighboring time series together according to the checks on the residuals of the time series. These residuals in spatial time series can provide meaningful patterns with neighboring locations, like showing how the traffic is evolving in the road network. The traffic flow time series dataset was tested, and the method can obtain better results than both the baseline and state-of-the-art methods. 

Wen et al. [82] presented a survey of the data augmentation methods that are specifically designed for handling time series datasets. Because many real-world applications like medical time series or anomaly detection time series face the problem of not having enough labeled data, it can be very helpful to have an effective way that can enlarge the size and improve the quality of the training data during deploying Deep Learning on time series datasets. Data augmentation has been found very suitable for this task, as it can generate data synthetically for unexplored input space with correct labels. Not only basic data augmentation methods, like time domain and frequency domain, but also advanced methods, like decomposition-based methods, statistical generative models, and deep generative models, are covered in the survey by Wen et al. These augmentation methods are found to be effective in time series classification, time series anomaly detection, and time series forecasting.

Azar et al. [83] developed a compression module for both univariate and multivariate time series data with the discrete wavelet transform and the error-bound compressor Squeeze (SZ). On-board processing and compression algorithms can reduce the problems associated with the transmission of large data volumes in applications of the Internet of Things (IoT). This preprocessing procedure is especially important for the sensor systems in IoT, as they may only have very limited bandwidth, memory, and computational power. The processed time series data is then sent to the Deep Learning models (Resnet, LSTM-FCN, GRU-FCN, FCN) for time series classification. The Fully Convolutional Network (FCN) deployed here consists of a convolutional layer with filters, a batch normalization layer, and then a ReLU activation layer. The experiment was conducted with time series datasets (UCR, UCI, and UEA) from sensors of ECG, motion, etc. The results supported that the compression approach can achieve a high compression ratio, while its time series denoising capability enables the Deep Learning module to achieve satisfactory classification accuracy.

## 3. Advances in Continual Learning Methods for Time Series Modeling

In the previous section, applications of Deep Learning models and preprocessing methods in different real-world time series scenarios have been reviewed. Limitations of Deep Learning models include a strict static requirement for the underlying process [92]. Post-deployment changes are not uncommon in the real world, but the DL methods are usually of fixed network structure after being deployed. When DL models are fed with data not following the independent and identically distributed (i.i.d.) assumption, destructive interference may occur and cause performance degradation [93]. Continual learning may be deployed to address these difficulties faced by DL models in such a way that Deep Learning models may benefit from continual learning to become capable of learning continually with adaptability. 

A major characteristic of continual learning is its sequential learning process. At each time, only a small amount of the input data is available. Other names for CL include lifelong learning, sequential learning, and incremental learning. Mathematically, the continual learning for both classification and time series regression problems can be expressed as follows [94]: Let $Τ=({Τ}_{1},\ldots ,{Τ}_{m})$ represent the *m* tasks that arrive in sequence. For task ${Τ}_{i}\left(i=1,2,\ldots ,m\right)$, there exist *N* instances of labeled time series data $D_{i}={\left\{\left(x_{i,r},y_{i,r}\right)\right\}}_{r\;=\;1}^{N}$. Here, time series $x_{i,r}\in {Χ}_{i}$ is associated with the corresponding target $y_{i,r}\in \Upsilon_{i}$. For classification tasks, the target space $\Upsilon_{i}$ refers to class labels. For time series regression tasks, the target space $\Upsilon_{i}$ refers to real numbers. A constraint of continual learning is that for any task ${Τ}_{i}$, there exists no access to the data of previous tasks ${Τ}_{j}\left(j=1,\ldots ,i-1\right)$. The common goal of each task is to learn a solver model $M_{i}$, such that $M_{i}:{Χ}_{i}\to \Upsilon_{i}$, with trainable parameters $\theta_{i}$ and estimated target ${\hat{y}}_{i,r}=M_{i}\left(x_{i,r};\theta_{i}\right)$. Let $L\left(y_{i,r},{\hat{y}}_{i,r};\theta_{i}\right)$ represent the training objectives. For classification problems, this can be the standard cross-entropy loss, while for time series regression problems, this can be the squared-error loss. Then,

$$L_{i}=\frac{q}{N}\sum_{r=1}^{N}L\left(y_{i,r},{\hat{y}}_{i,r};\theta_{i}\right)+\left(\frac{1-q}{\left(i-1\right)N}\right)\sum_{j=1}^{i-1}\sum_{r=1}^{N}L\left(y_{j,r},M_{i}\left({\hat{x}}_{j,r};\theta_{i}\right);\theta_{i}\right)$$

where *N* refers to the number of instances, and 0<q≤1 refers to the importance assigned to data from ${Τ}_{i}$.

There are several popular CL scenarios, like Instance-Incremental Learning (IIL), Domain-Incremental Learning (DIL), Task-Incremental Learning (TIL), Class-Incremental Learning (CIL), Task-Free Continual Learning (TFCL), and Online Continual Learning (OCL). This taxonomy is based on how the incremental batches are divided and which task identifies are available [18]. IIL refers to scenarios where all training samples arrive in batches and belong to the same task. DIL refers to scenarios where task identities are not needed; tasks have different input distributions but the same data label space. TIL refers to scenarios where task identities are available during both training and testing, and tasks have disjoint data label spaces. CIL refers to scenarios where task identities are available only during training and tasks have disjoint data label spaces. TFCL refers to scenarios where no task identity is available and tasks have disjoint data label spaces. OCL refers to scenarios where training samples for each task arrive from the data stream one by one, and tasks have disjoint data label spaces.

Flesch et al. [95] highlighted that for human continual learning, the extensive background statistical knowledge gained from previous unsupervised training might be utilized for rule learning at later stages, though with several conditions, while DNN suffers catastrophic forgetting (CF) problems. CF can be viewed as an issue stemming from the NN stability–plasticity dilemma, and the CF effect is associated with the abrupt loss of previously learned knowledge [17]. Plasticity refers to the NN’s ability to learn new knowledge, while stability means the storing of learned knowledge. CL can be employed in supervised learning, semi-supervised learning, unsupervised learning, and reinforcement learning as well. Shaheen et al. [96] discussed the applications of continual learning for autonomous systems. The real-world systems of vehicles with self-driving capability, unmanned aerial vehicles, and autonomous robotics were highlighted.

Various degrees of constraint simplification for solving the CF issue are imposed in the current methods. There are constraints such as memory, computational power, and data privacy. Nevertheless, there are issues when applying these methods, for example, too rigid constraints that may break the idea of learning continually or too tailor-made for solving a particular type of problem only. Pfulb and Gepperth [97] investigated the CF problems in DNN with a very large number of datasets in visual classification. The large-scale experiments show that no model and dataset is free of the CF problems, whilst a few potential workarounds may enable a few models to become practicable in a few application-level environments. Inspired by open-set recognition, Prabhu et al. [98] presented a model called GDumb, which is not tailor-made for CL tasks. GDumb starts with storing samples greedily in memory as they arrive, and during testing, it proceeds with new model training with samples only in the memory. GDumb is shown to outperform many CL approaches and may serve as an alert to the current progress in CL for classification, with the oversimplifications by some existing CL approaches to the problems resulting in little real-world application value. In the deep autoencoder NN for time series forecasting [99], buffers were deployed with online elastic weight consolidation to learn the probability distribution of the data stream sequentially. A CL model with explicit memory structure was used to address CF in their FFNN model for making long-term financial investment decisions [100]. Chen et al. [101] employed graph neural networks with CL strategies—data replay and parameter smoothing—for transferring learned knowledge to the current model in their traffic flow forecasting framework.

The relationship between neural network architectures and CF was investigated, and it was found that the network width has a significant effect on forgetting. Nevertheless, when increasing the width to address CF, inefficiency problems like long computation time and large energy waste inevitably appear. Lange et al. [17] investigated model capacity, weight decay, dropout regularization, and the stability–plasticity trade-off among continual learners. Lesort et al. [102] investigated the effects of the parameterizations of the DNN output layer on learning and forgetting in CL. The evaluation is conducted in a simplified learning environment, decomposing the model as a classifier part for the output layer and a feature extractor part for the rest of the DNN. Weights modifications, interference, and projection drift can be the causes of CF in the output layer. More knowledge is gained about the continual learning in output layers. Instead of focusing solely on continual learning, Mundt et al. [103] presented a framework combining concepts from CL, open set recognition, and active learning. This holistic approach shows promising results in addressing catastrophic forgetting and robustness for open-world applications.

Current limitations for CL applications include the predominant closed-world assumption when deploying models, which requires that any new data follows the same distribution as those used during training. When this closed-world assumption is no longer valid, the neural networks may give false predictions with unknown situations or with corrupted data [103]. Another limitation is that many current works in CL may not often consider the scalability issue in potential applications, which may have a very large number of sub-tasks and, consequently, huge amounts of samples. 

Whilst most of the recent works on CL focus on supervised tasks, the over-specializing of CL training for a single set of environments limits CL generalization to other types of applications [104]. With most recent research focusing on the CL applications for classification tasks, this lack of attention may hinder time series applications like renewable energy forecasting with CL models [99]. Deep Learning methods without the ability to remember old knowledge may not handle well in real-world applications of time series forecasting with non-stationary sequential data [105]. On the other hand, the current CL projects on different time series regression tasks are showing encouraging results. Pham et al. [105] designed a fast and slow learning network for online time series forecasting, with the per-layer adapter for fast learning and associative memory for remembering and recalling repeating past events. CL may be very useful for addressing financial time series problems, which can appear everywhere and are commonly non-stationary [100], and for traffic flow time series forecasting, which is essential for smart transportation. Most current methods of traffic flow forecasting assume static networks [101]. Yet, real-world traffic flow networks change constantly, like possible network modification and the addition of new parts [106].

**Figure 3:**
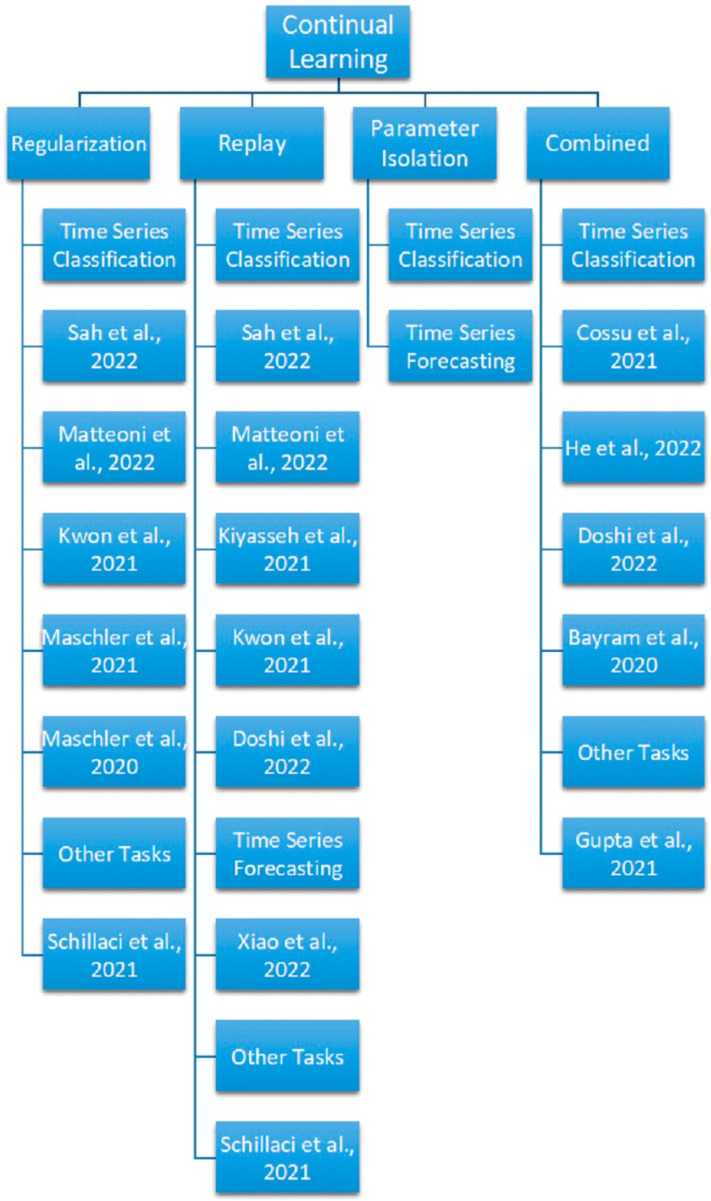
Tree diagram of the taxonomy of the continual learning methods for sensor time series classification and forecasting [93,94,106,107,108,109,110,111,112,113,114,115,116] (note: if a paper uses two methods separately with similar satisfactory results, the paper will be listed under both groups).

After the above discussion illustrates the importance of continual learning among the Deep Learning time series applications, a synthetic summary of the advances in continual learning techniques for time series applications is presented in Table 3, followed by a detailed description of the corresponding methods, motivations, accuracy, and brief details. The following Figure 3 shows the tree diagram of the taxonomy of the continual learning methods for sensor time series classification and forecasting.

As it is NP-Hard to find the optimal CL algorithms which can completely avoid catastrophic forgetting [117], polynomial time heuristic algorithms have been proposed to address the CF problems. These CL algorithms can be classified into three families, i.e., regularization-based methods, replay methods, and parameter isolation methods, according to the storage and usage method for task-specific information during the sequential learning process [17]. Combined approaches refer to the mixing of the methods together, and outstanding results can be obtained on benchmark datasets [103]. Another possible taxonomy is of regularization-based approach, replay-based approach, optimization-based approach, representation-based approach, and architecture-based approach [18]. This survey will follow the taxonomy of Lange et al. [17] and Mundt et al. [103].

### 3.1. Regularization-Based Methods

Regularization-based methods need to utilize an extra regularization term in the loss function for combining previous knowledge during the learning process of new data. Regularization methods focused on how to preserve previous knowledge. Extra loss terms can be deployed to preserve the important weights gained from the learning of former tasks. Elastic weight consolidation (EWC) [118], learning without forgetting (LWF) [119], and knowledge distillation [120] are popular regularization approaches. 

Sah et al. [107] investigated the performance of three recent continual learning approaches (A-GEM [121], ER-Ring [122], and MC-SGD [123]) for addressing the catastrophic forgetting in wearable sensors for activity recognition. In a wearable sensor system, it is necessary to have the capability to monitor and recognize activities across users. This sequential learning process is non-stationary and challenging for Deep Learning methods. The PAMAP2 dataset [124] of human activity recognition, consisting of sensor time series data of eight subjects and twelve daily activities, is used for the evaluation. The results showed that MC-SGD performed the best by reducing nearly 29% of the forgetting, while its computational time is still much less than the joint-task training time.

Matteoni et al. [108] developed two benchmarks of human state monitoring of domain-incremental scenarios for CL models. In non-stationary environments, recurrent neural networks for time series data can be of large importance [110], and investigation of the network properties in these situations may lead to a better understanding and applications of CL models. A significant obstacle that hinders the development of CL models is the shortage of enough standard benchmarks of time series datasets for the evaluation of the CL models. The two benchmarks were derived from datasets WESAD [125] and ASCERTAIN [126] of time series classification from physiological sequence data. Different CL models were investigated with these two benchmarks to understand the effects of catastrophic forgetting on recurrent neural networks. Four common CL models, i.e., replay [20], elastic weight consolidation (EWC) [118], learning without forgetting (LwF) [119], and naive and cumulative strategies, were tested. The results showed that all the CL models can mitigate forgetting, but besides the replay model, all other models still can not accumulate knowledge over time.

Kwon et al. [109] comprehensively investigated the performance of three CL approaches—regularization, replay, and replay with examples for deployment in mobile and embedded sensing devices. Most of the existing continual learning methods do not consider the resource requirements and limitations of mobile and embedded sensing devices [127]. Three datasets of human activity recognition, two datasets of gesture recognition, and one dataset of emotion recognition were investigated to find the trade-offs between system performance, storage requirements, computational power requirements, and peak memory requirements among the CL approaches. The results showed that the CL approach of replying with exemplars works best after considering all the trade-offs.

Maschler et al. [113] evaluated the performance of a continual learning approach based on regularization strategies, which mimic the human brain synaptic consolidation, for industrial application. Existing methods for anomaly detection often lack the flexibility to adapt to changes in the manufacturing processes [113], while continual learning can help overcome this issue by providing the automatic capability for adapting formerly learned knowledge to new settings. Real-world metal-forming time series dataset of a discrete manufacturing process was tested, showing promising results for the regularization strategies over the multilayer LSTM approach with no regularization. Online elastic weight consolidation is found to provide better performance than elastic weight consolidation. Maschler et al. [128] evaluated the performance of the regularization strategies against the open-access lithium-ion battery degradation dataset [129] with the LSTM approach for remaining useful lifetime prediction for lithium-ion batteries. The results of these degradation datasets reinforce the findings for the discrete manufacturing process. Network monitoring can produce a huge amount of multivariate time series data that are useful for usage, like network anomaly detection. Gonzalez et al. [130] outlined their conceptual framework of variational autoencoders and dilated convolutional networks for network anomaly detection. The deep generative replay is explored for extending the continual learning capability to the proposed system. The teacher generative model can create synthetic data to imitate previous training examples, while the new student model can learn from current new data and also these synthetic data at the same time. This continual learning module may help to address catastrophic forgetting.

Maschler et al. [114] proposed an LSTM algorithm with elastic weight consolidation for fault prediction in a distributed environment. Continual learning has the potential to serve as a distributed approach for fulfilling the industrial automation need, with Deep Learning performing fault prediction for industrial automation. This is because many real-world restrictions like industrial espionage and legal privacy concern prevent the centralizing of data from factories for the training of the Deep Learning models. NASA turbofan engine dataset [131] was used for performance evaluation. The experimental results showed that their approach could perform effectively with distributed datasets with no requirement for centralized data storage, satisfying the requirements of many real-world manufacturers.

Schillaci et al. [116] developed a continual learning RNN model with episodic memory replay and consolidation driven by prediction errors for transferring the knowledge gained from the greenhouse research facilities to real-world greenhouses. Without the CL capability, this process may otherwise be expensive and risky for the crop due to problems like the requirement of large-scale re-training in the new facility. Sensor time series data may help to better understand the causal models of a dynamic system, and this is very useful for real-world applications. Currently, popular causal discovery methods utilize only static data or pre-processed time series data in advance, making them not very suitable for real-world robotics cases. Continual learning may address this limitation in the causal discovery methods [132] but is under-investigated for robot application [133]. Castri et al. [133] focused on the constraint-based methods for causal discovery and outlined their approach of re-learning the causal model during observed scenario changes and during a new set of interventions. The new inference matric of the causal model is checked against the matric of the old causal model to discover the still valid causal links from the old model for the new model. This approach of only small incremental changes can help the robotic system address the CF problem. CL module can also help the robotic system address the hardware resource limitation. Sensors in a greenhouse can produce time series data like climate time series and leave temperature time series. A better understanding of the complex greenhouse and modeling of these greenhouse time series data can help to predict the effects of intervention better, thus increasing the crop yield. Memory retention is based on congruence against prior knowledge retained in the model, and the experimental results showed that this memory replay strategy could enhance the performance of standard memory consolidation approaches.

### 3.2. Replay Methods

Replay methods need to store either the raw samples or the generated pseudo-samples from the generative algorithm. These stored previous samples can be replayed to address forgetting during the learning of new tasks. Difficulties for this approach may include the demand for resources of large storage capability and the privacy concerns for storing and deploying samples in real-world applications. Pseudo-rehearsal techniques can handle these difficulties with a generative model instead of storing past samples directly. 

Kiyasseh et al. [93] utilized the replay buffer strategy to construct the continual learning model CLOPS from the temporal data in clinics, which is often non-stationary, as multiple clinical sites with various sensors are involved. Acquisition based on uncertainty was deployed for the replay of buffer instances. Four clinical environments involving transitions between data modalities, diseases, clinics, and time are suitable non-stationary scenarios to test the performance of the continual learning approach. The results show that CLOPS performs better in three scenarios than the two other frontier methods—GEM [134] and MIR [135]. He [111] also focused on the target-domain incremental application scenario and data-domain incremental application scenario of continual learning and described how their previous framework, CLeaR, can be extended to learn inputs successively. The framework utilizes the storage of buffered data by a novelty detector. The application of continual learning to the regression scenario for power forecasting was outlined.

Doshi and Yilmaz [112] proposed a new continual learning approach for video anomaly detection. Sensors and cameras in CCTV systems are generating huge amounts of real-time video temporal data, and data size may be too big to be investigated by humans. There is currently limited research on the continual learning of the new video data [112]. The CL capability was achieved in two ways, by the incremental updating of the memory module and by experience replay. A new, more comprehensive dataset was created, consisting of training segments in splits and test segments taken from a camera in a street in New Orleans, USA. Three existing benchmark datasets, i.e., UCSD Ped 2, CUHK Avenue, and ShanghaiTech, are also evaluated. The experimental results show that the proposed approach can perform excellently in overcoming practical VAD challenges.

Xiao et al. [106] have recently formulated the evolving long-term streaming traffic flow problem of sensor time series data as a continuous reinforcement learning task. With the advances in Deep Q-Networks (DQNs), which enable the learning of complicated reinforcement tasks, the research on reinforcement learning (RL) has grown enormously [136]. Agents in Deep RL have shown outstanding performance in settings with narrow tasks, but RL agents face problems like the over-fitting tendency and the lack of generalization capability to new variations [137]. This is in sharp contrast with the human ability to learn continually and be adaptive to new scenarios over a lifetime, which is called CL. DQNs face CF problems [118]. With the policy evolution leading to the non-stationary data distribution, the CL concept can be applied in RL [104]. In this way, CL methods may be used to address the CF, and solving this issue is crucial if the artificial agents are going to have the capability to learn continuously [138]. The continual learner has the advantage of adapting and recovering efficiently to changes encountered over time [29]. Here, the next flow value predictor is the agent, the next time series flow value in the sensor is the agent, and the dynamical sensor and transportation network is the environment state. The goal is to teach the autonomous agent to mimic sensor patterns and to plan the next visit according to the sensor profile. Prioritized experience replay strategy is deployed to transfer learned knowledge into the model, and KL divergence to utilize long-term pattern into model induction.

### 3.3. Parameter Isolation Methods

In parameter isolation methods, specific model parameters are assigned to each task such that possible forgetting can be avoided. The methods address the CF issues with the isolation of task-specific parameters and then dynamically adapt the structure of the model. New modules are deployed for the learning of new tasks while keeping the formerly learned parameters unchanged. A problem associated with this approach is the potential growth of network parameters required. Progressive networks [139] and dynamically expandable networks [140] are popular architectural methods. 

### 3.4. Combined Approaches

Combined approaches refer to the mixing of the methods together [103].

Cossu et al. [110] conducted a review and evaluation of the continual learning in RNNs, different from the typical CL focuses on feedforward and convolutional models. Sequential data is very popular in real-world applications like robotics. The sensors of the robotics system can feed the robot with time series input for its walk, learning in different environments. Yet, as the current focus of continual learning is on reinforcement learning through computer vision, there is not much research on sequential data processing for continual learning [110]. Two benchmarks of speech recognition and hand-drawn sketches were proposed for the evaluation of different CL methods. The results show that the forgetting issues become more serious for longer sequences.

He [111] presented the outline of their explainability module based on techniques like dimension reduction methods and visualization methods. Currently, there is not much research with a focus on the explainability of the continual learning algorithms [141], resulting in the problems like a lack of transparency for the CL modules. The module can not only provide the capability of explanation and visualization for the updated neural network of the CL module but also for the identified anomalies as well. The identification of anomalies can be achieved with machine learning approaches like the Deep Support Vector Data Description algorithm, unsupervised forest algorithm, and transformer-based unsupervised algorithm. The basic guideline is that novelties of scores higher than the threshold should be labeled as anomalies instead of labeling them as new tasks for the CL.

Bayram and Ince [115] presented a continual learning approach based on the Hidden Markov Model (HMM) for the auditory scene analysis (ASA) task. Sensor systems can be deployed to measure the time series data of dynamical acoustic events, and event recognition is about the identification of the events from these acoustic signals. High-value background noise and high computational demands restrict the deployment of continual learning approaches for this real-world ASA. A hierarchical HMM module is employed to evaluate acoustic event recognition and unknown event detection. The new knowledge gained from the HMM module is used for the retraining of a new HMM model in real-time in the continual learning module. Multiple acoustic events were evaluated with the proposed approach in real-time, and promising results of high effectiveness and high accuracy were obtained.

Gupta et al. [94] addressed the lack of practical variability among the industrial sensor networks by deploying an additional, conditional module to their generator-based RNN continual learning module. Real-time sensor time series data may be used for the in-process quality prediction by manufacturers. There are difficulties in limiting the applications of Deep Learning methods for quality prediction, with the main difficulties being the continuous changes in the manufacturing environments. In real-world applications of Deep Learning algorithms, various factories may have various settings, and their sensor environments may be different. Nevertheless, the current continual learning approaches do not consider the practical variability among the total numbers and types of sensors deployed in different manufacturing environments [94]. In injection molding, it is not uncommon to produce new products, and the pre-trained models cannot adapt to this new process well. Tercan et al. [142] developed a CL approach for this manufacturing scenario based on the memory-aware synapses method for the training of ANN with various manufacturing products. The experimental results of injection molding production support that the transfer of network weights gained from similar tasks can effectively raise the efficiency of the proposed approach. Graph neural network was employed in this conditioning module to control the deployment dynamics of their continual learning module such that the overall system can adapt to the different sensor settings in different real-world manufacturing environments [94]. Real-world datasets (DSADS [143], HAR [144], and Turbofan—FD001 [145]) were tested by randomly removing 40% of the sensors for each task. The experimental results support that their proposed conditioning GNN module can effectively enhance the capability of the continual learning module.

To summarize, promising results were obtained by the most recent research of CL applications with sensor time series data records for time series classification and time series forecasting tasks.

## 4. Conclusions

This survey has presented an overview of how to deploy Deep Learning methods, advanced preprocessing methods, and continual learning methods for time series classification and forecasting in different real-world practical scenarios. The review has summarized the encouraging results that CL can be deployed in fields beyond classification tasks. The non-stationary nature of the datasets in many fields poses challenges for conventional machine learning and Deep Learning while serving as good platforms for innovative CL applications in future works. Another very interesting direction is that further experiments may be done to assess the impact of architectures versus preprocessing algorithms such that the role of specific algorithms can be better understood. This is because, as observed in this survey, here, the majority of the authors focus on the practical deployment of DL and DL models solely, without further evaluating the influences of with and without preprocessing algorithms in each sensor environment.

## Figures and Tables

**Table 1:** Summary of the advances in DL techniques for sensor time series applications.

Ref.	First Author	Year	Application Field	Datasets	Deep Learning Models	Accuracy	Details
[32]	Choi	2021	Time series anomaly detection	Water treatment test-bed, water distribution pipelines, Mars Science Laboratory rover	RNN, CNN, hybrid, attention	No clear one-size-fits-all method	Compare DL anomaly detection time series models with benchmark datasets
[33]	Deng	2021	Detecting deviation from normal patterns	Sensor time series datasets of water treatment systems (SWaT and WADI)	Graph Deviation Network	54% better F-measure than the next best baseline	Combine graph neural networks with structured learning approach
[34]	Jiang	2020	Time series classification	UCR Time Series Classification Archive	MLP, CNN, ResNet	Not significantly better than 1-NN classifiers with dynamic time warping	Conduct comparison between nearest neighbor and DL models
[35]	Ismail Fawaz	2019	Time series classification	Univariate time series datasets of the UCR/UEA archive	MLP, CNN, Echo State Network	SOA performance achieved with CNN and deep Residual Networks	Conduct empirical study of DNNs for TSC
[36]	Han	2022	Leaf and wood terrestrial laser scanning time series classification	Seven broad-leaved trees (Ulmus americana) with a Rigel VZ-400i	Fully Convolutional Neural Network, LSTM-FCN, ResNet	Accurate separation of leaf and woody components from point clouds	Compare DL models on leaf and wood classification with a time series of geometric features
[37]	Campos-Taberner	2020	Classification of land use	Sentinel-2 time series data	2-layer Bi-LSTM network	Achieving best overall accuracy of 98.7%	Evaluate deep recurrent network 2-BiLSTM for land use classification
[38]	Naqvi	2020	Real-time classification of normal and abnormal driving	Database of driver facial emotion and gaze	CNN	Superior performance vs. previous methods	Apply CNN to find changes in gaze from driver’s images
[39]	Zheng	2020	Traffic flow time series forecasting	Traffic flow time series from OpenITS	LSTM	Outperform the ARIMA and BPNN	Deploy LSTM for traffic flow forecasting
[40]	Hua	2019	Traffic prediction and user mobility of telecommunication problems	Traffic time series	Random Connectivity LSTM	Reduced computing complexity by 30%	Deploy the Random Connectivity LSTM for traffic and user mobility prediction
[41]	Chen	2020	Equipment reliability prediction	Reliability test data of a cylinder in the small trolley of vehicle assembly plant	Deep Learning method based on MLP	Significant improvement over PCA and HMM	Employ DNN framework for reliability evaluation of cylinder
[42]	Lim	2021	Time series forecasting	M4 competition (Smyl, 2020)	Exponential smoothing RNN	Hybrid model with better performance than pure methods	Conduct survey of common encoders and decoders for time series forecasting
[43]	Yasrab	2021	Plant growth forecasting	Public datasets (Arabidopsis and Brassica rapa plants)	Generative Adversarial Network	Strong performance matching expert annotation	Employ generative adversarial predictive network for leaf and root predictive segmentation
[44]	El-Sappagh	2020	Alzheimer’s disease progression detection	Time series data from Alzheimer’s Disease Neuroimaging Initiative	Ensemble of stacked CNN and Bidirectional LSTM	Much better than conventional ML	Deploy deep network for detecting common patterns for classification and regression tasks
[45]	Lara-Benitez	2021	Twelve time series forecasting tasks	Twelve public datasets cover time series applications like finance, industry, solar energy, tourism, traffic, and internet traffic	MLP, Elman RNN, LSTM, Echo State Network, GRU, CNN, Temporal Convolutional Network	LSTM and CNN are the best choices	Evaluate seven popular DL models in terms of efficiency and accuracy
[46]	Cao	2020	Investigating temporal correlations of intra-series and the correlations of inter-series	Time series datasets from energy, electrocardiogram, and traffic sectors	Spectral Temporal Graph Neural Network	Outstanding forecasting results, plus advantage of interpretability	Develop the Spectral Temporal Graph Neural Network for multivariable time series forecasting
[30]	Rajagukguk	2020	Prediction of solar irradiance and photovoltaic	Time series data of temperature, humidity, and wind speed	RNN, LSTM, GRU, CNN-LSTM	Better prediction results than conventional ML	Evaluate models based on accuracy, forecasting horizon, training time, etc.
[47]	Torres	2018	Solar energy generation forecasting	Two-year time series of PV power from a rooftop PV plant	Deep Learning approach, based on the H20 package with the grid search method for hyper-parameter optimization	Particularly suitable for big solar data, given its strong computing behavior	Deploy DL approach for solar photovoltaic power forecasting for the next day
[48]	Xiao	2019	Prediction of sea surface temperature (SST)	SST time series data from 36-year observations by satellite	Convolutional Long Short-Term Memory	Outperform persistence model, SVR, and two LSTM models	Deploy ConvLSTM to capture correlations of SST across both space and time

**Table 2:** Summary of the importance of advanced preprocessing techniques for real-world sensor time series DL applications.

Ref.	First Author	Year	Application Field	Preprocessing Methods	Deep Learning Models	Accuracy	Details
[75]	Kanani	2020	ECG time series signals for monitoring and classification of cardiovascular health	Squeezing and stretching of the signal along the time axis	1D convolution	Achieved more than 99% accuracy	Develop a DL architecture for the preprocessing process for increased training stability
[76]	Kisa	2020	Surface electromyography time series of human muscles for gesture classification	Empirical mode decomposition	CNN	Worst results for original signal vs. all IMFs images	Deploy EMD to segmented signal to obtain the Intrinsic Mode Functions (IMFs) images for CNN
[8]	Zheng	2018	Classifying eight daily activities from wearable sensors	Segmentation and transformation methods	CNN	Achieved best results with multichannel method	Evaluate the impact of segmentation and transformation methods on DL models
[77]	Castro Filho	2020	Synthetic Aperture Radar images for rice crop detection	3D-Gamma filter and method of Savitzky and Golay	LSTM, Bidirectional LSTM	High accuracy and Kappa (>97%)	Apply 3D spatial–temporal filters and smoothing with Savitzky–Golay filter to minimize noise
[78]	ReBwurm	2020	Classifying crop type based on raw and preprocessed Sentinel 2 satellite time series data	Atmospheric correction, filtering of cloud temporal observations, focusing on vegetative periods, and masking of cloud	1D-convolutions, RNN, self-attention model	Preprocessing can increase classification performance for all models	Present the preprocessing pipeline, including atmospheric correction, temporal selection of cloud-free observations, cloud masking, etc.
[79]	Kingphai	2022	Classifying mental workload levels from EEG time series signals	Independent component analysis based on ADJUST	CNN, Stacked GRU, Bidirectional GRU, BGRU-GRU, LSTM, BiLSTM, BiLSTM-LSTM	Most effective model performance can be achieved	Deploy automatic ICA-ADJUST to remove the frequently contaminated artifacts components before applying DL models
[80]	Yokkampon	2022	Anomaly detection of multivariate sensor time series	Multi-scale attribute matrices	Multi-scale convolutional variational autoencoder	Achieved superior performance and robustness	Develop a new ERR-based threshold setting strategy to optimize anomaly detection performance
[58]	Barrera- Animas	2022	Rainfall prediction	Correlation matrix with the Pearson correlation coefficient	LSTM, Stacked-LSTM, Bidirectional LSTM	Retained the main features of DL models	Apply Pearson correlation matric for unsupervised feature selection
[81]	Mishra	2020	Wind predictions	Discrete wavelet transformation, fast Fourier Transformation, inverse transformation	Attention, DCN, DFF, RNN, LSTM	Performed best for attention and DCN with wavelet or FFT signal	Propose a preprocessing model of discrete wavelet transformation and fast Fourier transformation
[10]	Livieris	2020	Time series data from energy section, stock market, and cryptocurrency	Iterative transformations and Augmented Dickey–Fuller test	LSTM, CNN-LSTM	Considerably improved the DL forecasting performance	Propose transformation method for enforcement of stationarity of the time series
[1]	Asadi	2020	Traffic flow time series	Time series decomposition method	Convolution-LSTM	Outperformed SOA models	Deploy time series decomposition method for separating short-term, long-term, and spatial patterns
[82]	Wen	2021	Survey of data augmentation methods	Data augmentation methods (like time domain and frequency domain), decomposition-based methods, statistical generative models	Deep generative models	Show successes in time series tasks	Compare data augmentation methods for enhancing the quality of training data
[83]	Azar	2020	Wireless network with smart sensors	Discrete wavelet transform and the error-bound compressor Squeeze	Resnet, LSTM-FCN, GRU-FCN, FCN	Achieve the optimal trade-off between data compression and quality	Develop a compression approach with discrete wavelet transform and error-bound compressor

**Table 3:** Summary of the advances in continual learning techniques for time series applications.

Ref.	First Author	Year	Application Field	Motivations for Deploying CL	Continual Learning Models	Accuracy	Details
[107]	Sah	2022	Wearable sensors for activity recognition	Addressing the catastrophic forgetting in the non-stationary sequential learning process	A-GEM, ER-Ring, MC-SGD	Still need improvement for multitask training	Compare CL approaches for sensor systems
[108]	Matteoni	2022	Human state monitoring of domain- incremental scenario	Overcoming the non-stationary environments	Replay, elastic weight consolidation, learning without forgetting, naive and cumulative strategies	Existing strategies struggle to accumulate knowledge	Assess the ability of existing CL methods for knowledge accumulation over time
[93]	Kiyasseh	2021	Multiple clinics with various sensors for cardiac arrhythmia classification	Temporal data in clinics are often non-stationary	Buffer strategy to construct the continual learning model CLOPS	Outperform GEM and MIR	Apply uncertainty-based acquisition functions, for instance, replay
[109]	Kwon	2021	Deployment in mobile and embedded sensing devices	Addressing the resources requirements and limitations of the mobile and embedded sensing devices	CL approaches- regularization, replay and replay with examples	Best results for replay with exemplars schemes	Compare three main CL approaches for mobile and embedded sensing applications like activity recognition
[110]	Cossu	2021	Sensors of the robotics system	Achieving walk learning in different environments	Continual learning in RNNs	Highlight the importance of a clear specification	Evaluate CL approaches in class-incremental scenarios for speech recognition and sequence classification
[111]	He	2022	Identification of anomalies	Addressing the lack of transparency for CL modules	Explainability module based on dimension reduction methods and visualization methods	Proposed evaluation score based on metric	Propose the conceptual design of explainability module for CL techniques
[112]	Doshi	2022	Video anomaly detection (VAD)	Overcoming practical VAD challenges	Incremental updating of the memory module, experience replay	Outperform existing methods significantly	Develop a two-stage CL approach with feature embedding and *k*NN-based RNN model
[113]	Maschler	2021	Metal-forming time series dataset of a discrete manufacturing	Providing automatic capability for adapting formerly learned knowledge to new settings	Continual learning approach based on regularization strategies	Improved performance vs. no regularization	Compare CL approaches of regularization strategies on industrial metal-forming data for fault prediction
[114]	Maschler	2020	Fault prediction in a distributed environment	Real-world restrictions like industrial espionage and legal privacy concern prevent the centralizing of data from factories for the DL training	LSTM algorithm with elastic weight consolidation	Promising results for industrial automation scenarios	Apply elastic weight consolidation for distributed, cooperative learning
[115]	Bayram	2020	Auditory scene analysis	Addressing high-value background noise and high computational demands	Continual learning approach based on Hidden Markov Model	Achieve high accuracy	Develop an HMM-based CL approach with UED and retraining for time series classification
[106]	Xiao	2022	Evolving long-term streaming traffic flow	Addressing the non-stationary data distribution during policy evolution	Prioritized experience replay strategy for transferring learned knowledge into the model	Able to continuously learn and predict traffic flow over time	Formulate the traffic flow prediction problem as continuous reinforcement learning task
[116]	Schillaci	2021	Transferring the knowledge gained from the greenhouse research facilities to greenhouses	Addressing problems like the requirement of large-scale re-training in the new facility	Continual learning RNN model with episodic memory replay and consolidation	Outperform standard memory consolidation approaches	Present a CL approach of an episodic memory system and memory consolidation
[94]	Gupta	2021	In-process quality prediction by manufacturers	Addressing the lack of practical variability among industrial sensor networks	Generator-based RNN continual learning module	Possible significant performance enhancement	Deploy task-specific generative models to augment data for target tasks

## Data Availability

Data sharing is not applicable to this article as no new data were created or analyzed in this study.
